# Market access pathways for cell therapies in France

**DOI:** 10.3402/jmahp.v3.29094

**Published:** 2015-11-18

**Authors:** Cécile Rémuzat, Mondher Toumi, Jesper Jørgensen, Panos Kefalas

**Affiliations:** 1 Creativ-Ceutical, Paris, France; 2 Laboratoire de Santé Publique, Faculté de Médecine, Aix-Marseille Université, Université de la Méditerranée, Marseille Cedex, France; 3 Cell Therapy Catapult Limited, London, UK

**Keywords:** France, advanced therapy medicinal products, cell therapy, pricing, reimbursement, market access, funding

## Abstract

**Introduction and objective:**

Cell therapies can be classified into three main categories of products: advanced therapy medicinal products (ATMPs), ATMPs prepared on a non-routine basis (hospital exemptions), and minimally manipulated cells. Despite the benefits that cell therapies can bring to patients, they are subject to complex pathways to reach the market in France. The objective of this study was to identify and describe routes to market access for cell therapies in France and how these vary by regulatory status.

**Methodology:**

The research was structured following five main steps: (1) identification of the French regulatory framework for cell therapies; (2) identification of the health products categorised as cell therapies in France; (3) mapping of the market access pathways per category of cell therapy; (4) validation of findings by interviewing experts; and (5) development of a roadmap summarising market access pathways for cell therapies in France. The secondary research methodology included a comprehensive literature review conducted on websites of French public health institutions, complemented by a research for peer-reviewed articles, abstracts, and grey literature.

**Results:**

Different market access pathways are possible depending on the cell therapy category. For ATMPs, market access pathways depend on the licensing status of the therapy. Licensed ATMPs followed the same market access pathways as ‘conventional’ pharmaceuticals, whereas not-yet-licensed ATMPs can be funded via a specific financial allowance under the framework of a Temporary Authorisation for Use procedure or various research programmes. For new ATMPs that are associated with a separate medical device (not considered as ‘combined ATMPs’) or associated with a new medical procedure, additional pathways will apply for the medical device and/or medical procedure to be reimbursed in the ambulatory settings or at hospital. The most likely funding option for ATMPs prepared on a non-routine basis is outside the diagnosis-related group (DRG) system through Missions of General Interest and Support to Contracting (MIGAC). For minimally manipulated cells, four different funding processes are applicable, depending on the type of activity: (1) inclusion in a DRG; (2) inclusion in the list of products and services qualifying for reimbursement (LPPR) (as a medical device); (3) an annual lump sum provided by regional health agencies; and (4) a financial allowance under Missions of General Interest (MIG).

**Conclusion:**

Cell therapy is a diverse and promising category of medical interventions. Its heterogeneity and complexity mean that several funding options and market access pathways apply. The main challenges facing cell therapies relate to (1) the identification of the most appropriate path to reimbursement, and (2) price setting, whereas high manufacturing costs of these therapies will dictate a high price that could only be achieved by a product that leads to important additional patient benefits compared to available treatment options. More specific funding options could emerge as the number of cell therapies increases and the authorities face the need to structure and stabilise funding. It will be vital for manufacturers to have a clear understanding of the various temporary funding opportunities early in a product's lifecycle for the adoption of a stepwise approach to secure permanent funding. Furthermore, due to the very limited Health Technology Assessment (HTA) bodies experience for cell therapies, manufacturers should enter into dialogues with HTA agencies at an early stage to optimise market access conditions.

Recent advances in medicine and biological research have spurred the development of cell therapies for the treatment of various diseases. Cell therapy is defined by the American Society of Gene and Cell Therapy as ‘the administration of live whole cells or maturation of a specific cell population in a patient for the treatment of a disease’ (1).

## European regulations related to cell therapies

In Europe, the Directive 2004/23/EC (2) set standards of quality and safety for the donation, procurement, testing, processing, preservation, storage, and distribution of human tissues and cells.^1^ For tissues and cells intended to be used for industrially manufactured products, further manufacturing steps are covered by Directive 2001/83/EC relating to medicinal products for human use (3). Due to the technical specificities and complexity of such therapies, harmonised rules were introduced in 2008 (European Commission (EC) Regulation No. 1394/2007 (4)) for the marketing of the so-called advanced therapy medicinal products (ATMPs), amending Directive 2001/83/EC and specifying the rules concerning their marketing authorisation, supervision, and monitoring.

Cell therapies can be classified into three main categories of products: ATMPs, ATMPs prepared on a non-routine basis (hospital exemptions), and minimally manipulated cells.

### Advanced therapy medicinal products

In Europe, the Human Tissue Authority defines ATMPs as ‘innovative, regenerative therapies which combine aspects of medicine, cell biology, science and engineering for the purpose of regenerating, repairing or replacing damaged tissues or cells’ (5), a definition that encompasses gene therapy medicinal products, somatic cell therapy medicinal products,^2^ tissue-engineered products,^3^ and combined ATMPs^4^ (3, 4, 6). The main difference between somatic cell therapy medicinal products and tissue-engineered products is the intended function of the product (i.e., the prevention, diagnosis, and/or treatment of diseases via pharmacological or metabolic actions for somatic cell therapies versus actions to regenerate, repair, or replace human tissue for tissue engineered products) (6). A cell therapy is considered as a medicinal product if it has the following attributes: it consists of the cells or tissues that have been subject to substantial cell or tissue manipulation and/or is intended for non-homologous use (i.e., cells or tissues are not intended for use for the same essential function(s) in recipient and donor) (4).

The ATMP regulation was designed to ensure a high level of human health protection and a free movement of ATMPs within the European Union (EU) (7). These products are authorised through a centralised marketing authorisation process and are assessed by a specialised body (the Committee for Advanced Therapies, or CAT). Between 2009 and May 2014, 12 marketing authorisation applications for ATMPs were filed, from which only four were approved. Among these, three were cell-based medicinal products which include both somatic cell therapy medicinal products and tissue engineered products (8). An additional cell therapy product was approved in February 2015 (9).

### ATMPs prepared on a non-routine basis (hospital exemptions)

ATMP regulation includes a specific exemption to this marketing authorisation, the so-called hospital exemption, which provides the EU Member States with the power to authorise the use of ATMPs prepared on a non-routine basis, provided that the product is used for individual patients in a hospital and under the professional responsibility of a medical practitioner (7). Some concerns have been raised with regard to this non-routine authorisation for ATMPs, due to differences in available efficacy and safety data compared to those for licensed equivalents; therefore, these exemptions are closely monitored by the EC (7).

### Minimally manipulated cells

Unlike ATMPs and hospital exemptions, minimally manipulated cells are not medicinal products. They are characterised by the two following criteria: no substantial cell or tissue modification, and the intention for homologous use (i.e., used in the same essential function(s) in recipient and donor) (6).

There is variation in the degree of manipulation associated with the term ‘minimally manipulated’, as exemplified by simple cell separation approaches (e.g., platelets used in transfusions to prevent bleeding in patients with thrombocytopenia) compared to more complex selection processes (e.g., Cytovir CMV for the prevention of cytomegalovirus reactivation in patients following haematopoietic stem cell transplantation).

## French context for market access of cell therapies

In France, the different categories of cell therapies (i.e., ATMPs, hospital exemptions, and minimally manipulated cells) are governed by different regulatory statuses, which in turn lead to different market access pathways (10). So far, only one ATMP (ChondroCelect^®^) has been assessed by the Transparency Committee (Commission de la Transparence, or CT), the French health technology assessment agency, but reimbursement was not granted (11, 12).

These therapies are facing several challenges in terms of market access in France. Although cell therapies have the potential to cure chronic and/or severe diseases, long-term claims at launch are unlikely to be substantiated by data generated directly from pre-launch randomised clinical trials, meaning modelled data and extrapolations are likely to be leveraged by manufacturers to support such claims. Furthermore, some concerns have arisen around the potential budget impact on the healthcare system, especially in the short term. In the current context of pressured healthcare budgets, access to these therapies will be highly scrutinised by payers, and mechanisms addressing uncertainty are likely to be sought. In recent years, France has introduced a set of laws to control drug expenditure, including but not limited to price cuts, generic incentives, and health economic assessment (13). In addition to that, market access for innovative therapies in France is characterised by the fragmentation and complexity of its funding mechanisms, which include several lists for reimbursable medicines, various funding options outside the diagnosis-related group (DRG) system, and funding opportunities for early access of innovative therapies. Cell therapy is also a new and innovative field in which both the pharmaceutical industry and payers have little experience. Despite the benefits cell therapies can bring to patients, they are subject to complex pathways to reach the market in France which involve various influencers, decision makers, and assessment criteria. It is therefore important for the cell therapy industry to understand the specificities of the French market access processes in order to plan and secure therapy adoption.

The objective of this study was to identify and describe routes to market access for cell therapies in France and how these vary by regulatory status.

This article provides an overview of the French regulatory framework for cell therapies and a mapping of the French market access pathways per category of cell therapy; key challenges related to these pathways are addressed, and strategic recommendations are provided to manufacturers.

## Methodology

The research was based on a comprehensive literature review conducted on websites of French public health institutions – the Ministry of Health,^5^ the National Agency for the Safety of Medicine and Health Products^6^ (Agence Nationale de Sécurité du Médicament et des produits de Santé, or ANSM), the French National Authority for Health^7^ (Haute Autorité de Santé, or HAS), the National Health Insurance,^8^ and the Biomedicine Agency^9^ – to identify the French regulatory framework for cell therapies and the different funding options per category of cell therapy; related French regulation was identified through the official website of the Public Health Code (PHC) and Social Security Code (Legifrance^10^). This review was complemented by research for peer-reviewed articles on MEDLINE and relevant recent International Society for Pharmacoeconomics and Outcomes Research conferences. Additional internet searches using Google and Google Scholar were also conducted. Grey literature was also searched for, following experts’ inputs.

The search strategy consisted of the following free search terms: ‘cell therapy’, ‘ATMPs’, ‘ATMPs prepared on a non-routine basis’, ‘cell preparations’, ‘hospital exemption’, ‘pricing’, ‘price’, ‘reimbursement’, ‘funding’, ‘market access’, and ‘France’. The search was conducted in the French and English languages. There was no limitation in terms of publication dates. The cut-off date for search was May 2015.

This research was structured following five main steps:

1. First, we identified the French regulatory framework for cell therapies, including regulatory categories and marketing authorisation processes.
2. We subsequently identified the health products categorised as cell therapies in France.
3. Then, we proceeded to map the market access pathways per category of cell therapy, including the key stakeholders involved at national, regional, and local levels and the decision analysis framework for pricing, reimbursement, and market access. When no specific information was available for a certain product category, the most appropriate pathway was assumed based on the existing funding options in France considering the relevant product characteristics.
4. These findings were validated by interviewing experts in the field of market access of health products in France. A discussion guide was developed to support the standardisation of the interview (see Supplementary file). The experts’ interviews covered the French approval and funding process for cell therapies, as well as current and future challenges in this field.
5. Finally, the various funding pathways were summarised in a roadmap to allow an overview of different cell therapy categories, market access processes, key stakeholders, and their interrelationships.

## Results

### Identification of a national regulatory framework for cell therapies

In France, cell therapies are classified in the following three categories (10, 14):

- ATMPs (also known as Médicaments de Thérapie Innovante, or MTIs), including gene therapy medicinal products, somatic cell therapy medicinal products, tissue engineered products, and combined ATMPs (with a medical device). For these products, marketing authorisation is regulated at the EU level through a European Medicines Agency (EMA) opinion followed by an EU commission decision, and then transposed at the national level (‘blue box’^11^). For combined ATMPs, marketing authorisation must include evidence of conformity with essential requirements and the notified body, in conformity with the directives related to medical devices (Directives 93/42/EEC (15) and 90/385/EEC (16) (as amended)) (10).
- ATMPs prepared on a non-routine basis (also known as Médicaments de thérapie innovante préparés ponctuellement, or MTI-PPs), defined as ATMPs used within the same EU Member State, in a hospital, under the responsibility of a physician, and in accordance with a medical prescription for an individual patient (‘hospital exemption’). For these products, marketing authorisation is regulated at the national level through the ANSM.
- Cell preparations (‘Préparations cellulaires’), referring to products that are not considered as ATMPs but contain human tissues or cells that are minimally manipulated and intended for homologous use. For these products, marketing authorisation is regulated at the national level based on transposition of EU Directive 2004/23/EC (2) related to standards of quality and safety for the donation, procurement, testing, processing, preservation, storage, and distribution of human tissues and cells.

Definitions and comparative regulatory frameworks for the different categories of cell therapies in France are summarised in Table 1.

**Table 1:** Definitions and comparative regulatory framework for the different categories of cell therapies in France (10, 14)

	ATMPs	ATMPs prepared on a non-routine basis	Minimally manipulated cells
French terminology	Médicaments de Thérapie Innovante (MTI)	Médicaments de thérapie innovante préparés ponctuellement (MTI-PP)	Préparation cellulaire
Product category	Medicinal product	Medicinal product	Not a medicinal product
Definition	Gene therapy medicinal products, somatic cell therapy medicinal product, tissue engineered product, combined ATMP (with medical device)	ATMPs prepared on a non-routine basis and used within the same EU Member State in a hospital in accordance with a medical prescription for an individual patient (‘hospital exemption’)	Products not considered as ATMPs but containing minimally manipulated human tissues or cells intended for homologous use
Marketing authorisation	• EU centralised marketing authorisation (European Directive 2001/83/EC (amended) & Regulation No. 1394/2007) • Paediatric regulation 1901/2006/EC applicable	• National marketing authorisation (ANSM) (French Law No. 2011-302, Decree No. 2012-1236 & Decision of 04/02/2013) • Paediatric regulation 1901/2006/EC not applicable	• National marketing authorisation (ANSM) (French law No. 2004-800/2011-814 (article L.1243-1 et seq. of public health code)) • Paediatric regulation 1901/2006/EC not applicable
Manufacturing establishment	Pharmaceutical establishment (public or private) (Articles L.5124-9-1 and L.5124-1 PHC)	• Pharmaceutical establishment (public or private) (Articles L.5124-9-1 and L.5124-1 PHC) • Other establishments authorised by ANSM with opinion of Biomedicine Agency (article L.4211-9-1 PHC)	Cell therapy unit authorised by ANSM (Article L.1243-2 PHC)
Good manufacturing practice (GMP)	GMP for medicines (Article L.5121-5 PHC)	• Pharmaceutical establishment: GMP for medicines • Other establishments authorised by ANSM: specific GMP guidance to be published (Article L.5121-5 PHC)	GP ‘tissues/cells’ (Article L.1245-6 PHC)
Clinical trials	• Directive 2001/20/EC^a^ • Law No. 2004-806, application decree 2006-477, and decisions related to medicines^b^	• Directive 2001/20/EC^a^ • Law No. 2004-806, application decree 2006-477, and decisions related to medicines^b^	• Law No. 2004-806, application decree 2006-477 and decisions related to cell therapy preparation^b^ (directive 2001/20/EC not applicable)
Vigilance	Pharmacovigilance	Pharmacovigilance	Biovigilance (Article L.1211-7 PHC)
Import/export	Possible	Not possible	Possible

PHC: Public Health Code; ANSM: French National Agency of Medicine and Health Products Safety.

a On 16 April 2014, the new regulation No. 536/2014 of the European Parliament and of the Council on clinical trials on medicinal products for human use, and repealing directive 2001/20/EC, was adopted, and published in the official journal on 27 May 2014. It entered into force on 16 June 2014 but will apply no earlier than 28 May 2016.

b Law 2012-300 on clinical trials adopted in March 2012, but application decrees have not yet been published.

### Market access pathways for cell therapies in France

#### ATMPs

ATMPs are by definition medicinal products and, as such, follow the same market access pathways as ‘conventional’ pharmaceuticals. Combined ATMPs (i.e., ATMPs used in combination with a medical device), in which the medical device is considered an integral part of the final product (6), also go through the same pricing and reimbursement pathways as pharmaceuticals.

However, several market access pathways are possible depending on the licensing status of the therapy (i.e., licensed ATMPs or not-yet-licensed ATMPs). For new ATMPs that are associated with a separate medical device (and not considered as ‘combined ATMPs’) or associated with a new medical procedure, additional pathways will apply for the medical device and/or medical procedure to be reimbursed in ambulatory settings or at hospital.

##### Licensed ATMPs

**Fig. 1:**
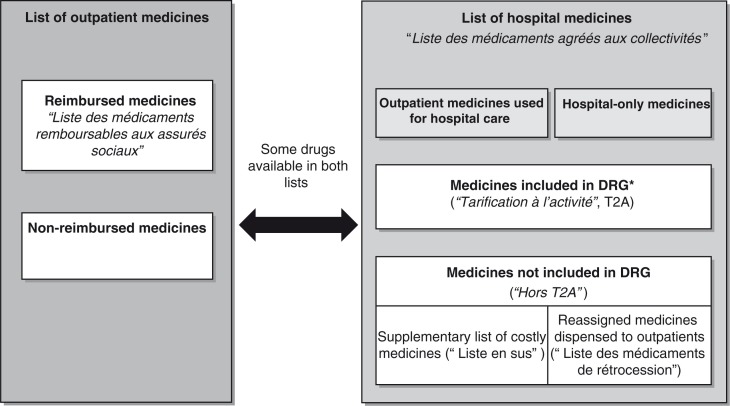
French listing of medicines (17–19). *About 40% of medicines used in hospital. DRG: diagnosis-related group.

**Fig. 2:**
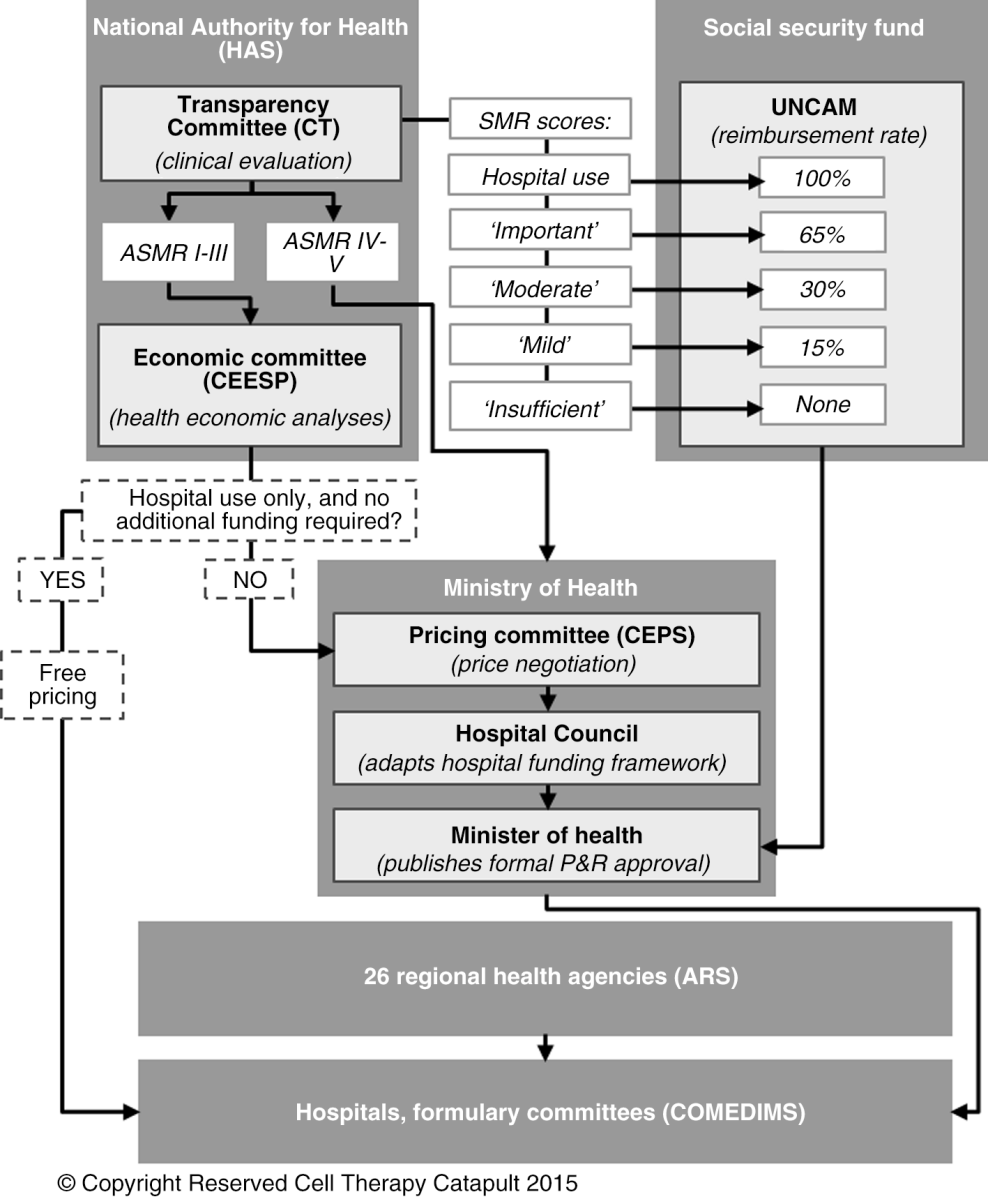
Market access process for drugs in France. ARS: Regional Health Agencies; COMEDIMS: Committee for Medicines and Sterile Medical Devices; CEESP: Economic and Public Health Assessment Committee; CEPS: Economic Committee for Health Care Products; CT: Transparency Committee; HAS: French National Authority for Health; P&R: pricing and reimbursement; SMR: actual benefit; ASMR: improvement in actual benefit; UNCAM: National Union of Health Insurers.

Pricing and reimbursement decisions for licensed ATMPs are centralised and based on technical opinion from the HAS through the CT and the Economic and Public Health Assessment Committee (Commission Evaluation Economique et de Santé Publique, or CEESP). The final decision to include the drug on the list of reimbursed medicines or in the hospital list of medicines lies with the Ministry of Health. Price setting depends on the list on which the drug is included. For reimbursed outpatient medicines, prices are negotiated with the Economic Committee for Healthcare Products (Comité Economique des Produits de Santé, or CEPS). For hospital medicines included in the reassigned medicines (‘médicaments de rétrocession’) list and supplementary list (‘Liste en sus’), a ceiling price for reimbursement is set by the CEPS. For other medicines that are included and covered by the DRGs, manufacturers can set the price freely (Fig. 1) (13, 17–19). The CEPS uses the CT (and the CEESP where applicable) opinions to negotiate the price with manufacturers. Following pricing and reimbursement decisions, outpatient medicines can be made available in retail pharmacies, whereas inpatient medicines have to be included in hospital formularies based on an assessment performed within each hospital by the Committee for Medicines and Sterile Medical Devices (Commission du Médicament et des Dispositifs Médicaux Stériles, or COMEDIMS). The decision is mainly based on hospital needs, taking into account the therapeutic value and the costs (17). Drug access is then indirectly regulated at the regional level by the Regional Health Agencies (Agences Régionales de Santé, or ARS), which are responsible for regional implementation of national health policies, (among other missions) monitoring of prescriptions by hospital practitioners of medicines dispensed to outpatients (Prescriptions Hospitalières Médicamenteuses Exécutées en Ville, or PHMEV), as well as monitoring of prescriptions of costly medicines included in the supplementary list (‘Liste en sus’) (20) (Fig. 2).

During drug evaluation, the CT assesses the actual benefit (Service Médical Rendu, SMR), which determines the reimbursement rate, as well as the improvement in actual benefit (Amélioration du Service Médical Rendu, ASMR), which is used in the price negotiation along with the target population in price–volume agreements (Fig. 2). The SMR is set based on the severity of the disease, the efficacy (effect size) and safety, the position of the treatment in the therapeutic strategy, the impact on public health, and the type of treatment (preventive, curative, or symptomatic). There are five levels of SMR (major, important, moderate, weak, and insufficient) that drive the reimbursement rates set by the National Union of Health Insurance Funds (Union Nationale des Caisses d'Assurance Maladie, UNCAM): 100% or 65%, 65%, 30%, 15%, and 0%, respectively. The ASMR is set based on the assessment versus relevant comparators by indication and/or therapeutic strategy. The improvement in benefit (ASMR score) is mainly driven by the effect size of the incremental clinical efficacy benefit. Safety and quality of life are considered, especially if the clinical disease burden is substantial. There are five ASMR levels (I: major improvement; II: substantial improvement; III: moderate improvement; IV: minor improvement; and V: no improvement) driving price negotiations with the CEPS (13, 17–19). An ASMR I, II, or III^12^ level allows a fast-track pricing process (about 3 weeks, instead of a 3-month process) with international reference pricing based on the four large EU countries (Germany, Italy, Spain, and the United Kingdom), along with a 5-year EU price guarantee. An ASMR IV can lead to a slight price premium over comparators provided it does not lead to high budget impact, whereas an ASMR V must be associated with lower prices than those of relevant comparators to decrease budget impact. Other pricing drivers include competitors’ prices, EU prices already obtained in the four large EU countries, target population size, budget impact, health economics for innovative drugs, as well as the French financial context and the footprint of the manufacturer in France.^13^ In practice, there are often important differences between the net price for the manufacturer and the published price, as confidential pricing agreements are often agreed between the manufacturer and the CEPS (17, 18, 21). For innovative medicines for which there is an anticipated but unproven benefit, a conditional price might be granted under very strict conditions. The price is conditional on results of a study (coverage with evidence development) to assess the drug benefit, and it is reassessed in the short or medium term (maximum 3 years). In case of study failure, drug price cuts and/or paybacks will be imposed on the manufacturer (21).

The CT assessment is also considered, among other criteria,^14^ when the Hospital Council decides on inclusion of costly medicines in the ‘Liste en sus’. The Hospital Council is an organisation that includes representatives from central administrative Directorates of the Ministry of Health and national health insurance funds, which are responsible for advising the Ministry of Health on hospital funding frameworks. The final decision for inclusion in the ‘Liste en sus’ is taken by the Ministry of Health. A drug awarded an ASMR I to III is a positive argument for inclusion in the ‘Liste en sus’, whereas drugs awarded ASMR IV or V could be considered for inclusion in this list if the drug has been compared directly to another product included in the ‘Liste en sus’; in cases where there is no direct comparator, other pertinent comparators will be analysed, depending on their place in the therapeutic strategy, ASMR level, and funding modalities (22) (Fig. 2).

The CEESP is in charge of health economic assessment for innovative products. Since October 2013, pharmaceutical companies are required to file a health economic dossier to the CEESP for drugs that claim an ASMR I to III *and* have a significant impact on the health insurance budget (i.e., expected to generate more than €20 million in annual sales revenue during the second full year on the market). The CEESP dossier filing is performed in parallel to the CT dossier filing, and the two committees perform their assessment in parallel (Fig. 2). The CEESP opinion aims to validate the incremental cost-effectiveness ratio (ICER) associated with each drug versus its most relevant comparator. Its opinion relies on conformity of health economic evaluations with the guideline related to methodological choices for economic assessment issued by the HAS in 2011 (23). No ICER threshold has been defined in France, and the CEESP is not expected to be prescriptive in this respect. The CEESP opinion is forwarded to the CEPS for price setting and mainly used to determine a potential rebate on the price set via international reference pricing. The CEESP opinion is made publically available once the price negotiation is concluded (13, 24, 25).

##### Medical devices and procedures

Following the introduction of the ATMP regulation by EMA, cell therapies cannot be classified as medical devices. However, medical devices may be used in combination with ATMPs, and in this context they are discussed here. Pricing and reimbursement of medical devices bear some similarities to those of drugs. Technical advice is also given by the HAS but through the National Committee for the Evaluation of Medical Devices and Health Technologies (Commission Nationale d'Evaluation des Dispositifs Médicaux et des Technologies de Santé, or CNEDiMTS). Once the CE mark is obtained, different methods can be used to apply for market access of a medical device. To be reimbursed in the outpatient sector, a medical device has to be included on the list of products and services qualifying for reimbursement (Liste des Produits et Prestations Remboursables, or LPPR). There are two pathways for inclusion of a medical device on the LPPR: either under generic description, meaning that the product is identified according to its indications and technical specifications (auto-registration on LPPR by the manufacturer, i.e., no preliminary CNEDiMTS assessment, and a similar reimbursement price as those of products under the same generic description^15^), or under brand name for innovative products or those requiring specific monitoring^16^ (CNEDiMTS assessment for LPPR inclusion). In the hospital setting, medical devices can be included either in the DRG or in the LPPR and thus funded on top of the DRG tariff. Of note, the HAS can assess some medical devices included in DRG tariffs. Similarly as for drugs, the COMEDIMS is in charge of defining the medical devices to be included on the hospital formulary (26, 27).

The CNEDiMTS assesses the actual benefit (Service Attendu, or SA) and the added clinical value (Amélioration du Service Attendu, or ASA). The SA is based on the risk–benefit ratio, the position in the therapeutic strategy, and the impact on public health. If the SA is assessed as insufficient, the medical device will not be included in the LPPR (no reimbursement). If the SA is considered as sufficient, the CNEDiMTS will assess the ASA per indication versus relevant comparators (product, procedure, or services). Similarly as for drugs, there are five ASA levels (I, major improvement; II, substantial improvement; III, moderate improvement; IV, minor improvement; and V, no improvement). The decision to include the device on the LPPR lies with the Ministry of Health after the CNEDiMTS has given its guidance. The medical device reimbursement tariff is then negotiated between the CEPS and the manufacturer based on the SA, the ASA, the tariffs and prices of comparators included on the LPPR, the volume of anticipated sales, as well as the anticipated pattern of use at time of launch.

Similarly as for drugs, the CEESP is in charge of health economic assessment for innovative medical devices (i.e., medical devices for which the company claims an ASA I to III and that have a significant impact on the health insurance budget, i.e., expected to generate more than €20 million in annual sales revenue during the second full year on the market) (23, 24).

**Fig. 3:**
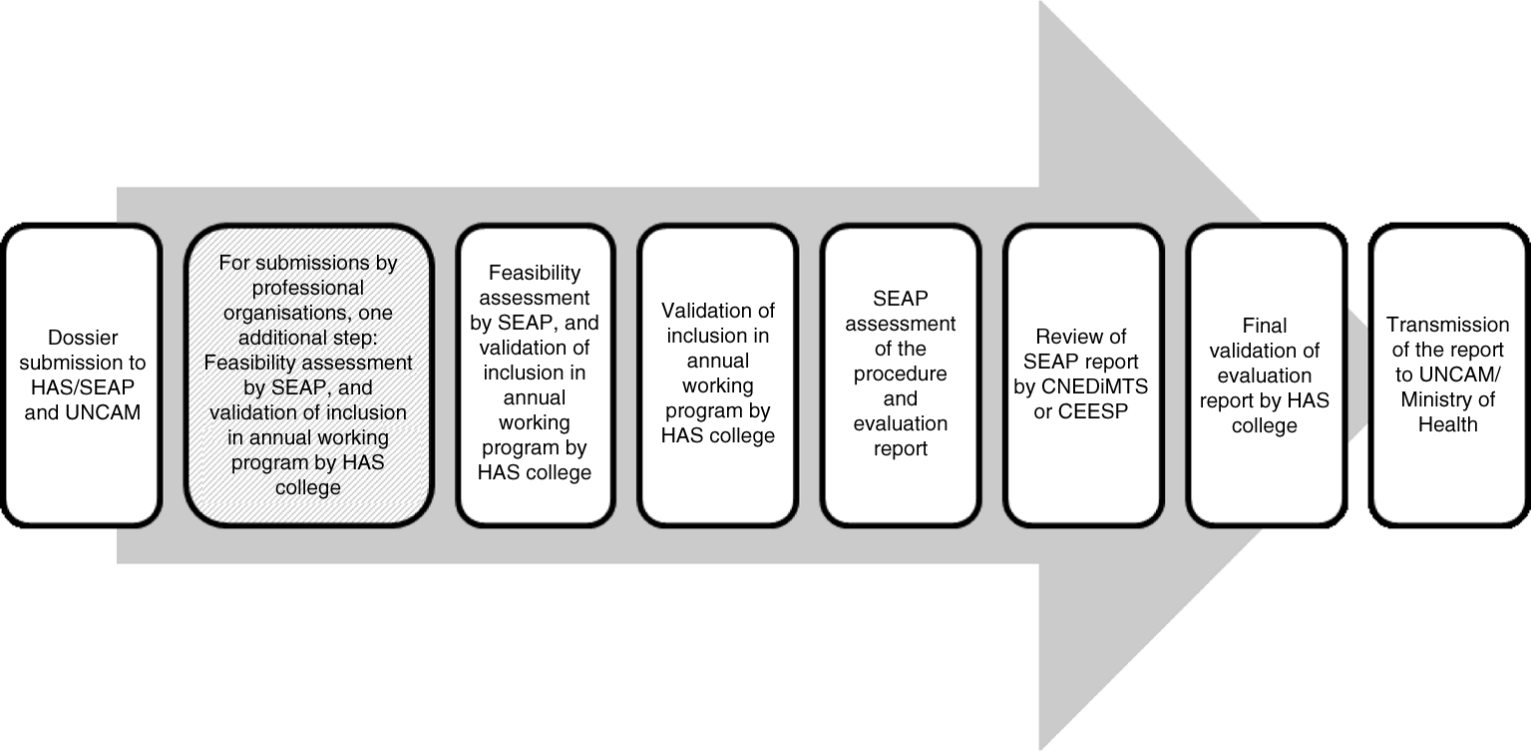
Medical procedure assessment process (27–29). CEESP: Economic and Public Health Assessment Committee; HAS: French National Authority for Health; DGOS: General Directorate of Health Care Supply; DGS: Health General Direction; DSS: Social Security Direction; SEAP: Department of Medical Procedures Assessment; UNCAM: National Union of Health Insurers.

New medical procedures are reimbursed through inclusion in the DRG tariffs (i.e., by DRG re-assessment to include the procedure in existing tariffs), by setting up a new DRG, or by inclusion in the medical procedure nomenclature (specific classification) for procedures in the private sector (ambulatory care or hospital). Depending on their nature, medical procedures can be included in the general nomenclature of medical procedures (Nomenclature Générale des Actes Professionnels, or NGAP; e.g., dental implants), the joint classification of medical procedures (Classification Commune des Actes Médicaux, or CCAM; e.g., osteodensitometry for the diagnosis of osteoporosis), or the nomenclature of procedures in laboratory medicine (Nomenclature des Actes de Biologie Médicale, or NABM; e.g., researching HIV or hepatitis C virus in sperm). The Department of Medical Procedures Assessment (Service d'Evaluation des Actes Professionnels, or SEAP) of the HAS gives its guidance on the possibility of including medical procedures in the different nomenclatures (CCAM, NABM, or NGAP). The final decision is taken by the UNCAM and Ministry of Health. The reimbursed price is not negotiated; it is set by the Ministry of Health. The request for inclusion in the nomenclature can be carried out by the UNCAM, the Ministry of Health, the HAS, a professional organisation (e.g., scientific societies), or a validated users’ association (26, 28, 29). The approval process for medical procedures is summarised in Fig. 3.

##### Not-yet-licensed or -launched ATMPs

Several funding options are available in France to grant early access to innovative therapies.

Medicinal products that have not yet been granted a marketing authorisation and that are not currently available through clinical trials in France can gain access to the French market through the Temporary Authorisation for Use procedure (Autorisation Temporaire d'Utilisation, or ATU). The ATUs have existed since 1994 and are granted by the ANSM on an exceptional and temporary basis. The aim of this procedure is to treat, prevent, or diagnose serious or rare pathologies in the absence of a suitable therapeutic alternative available in France, when the risk–benefit ratio of the medicinal product is considered likely to be positive by ANSM. The use of ATUs improves time to market for new treatments by an estimated 25 months on average compared to standard licensing (30–32).

Two types of ATUs exist: (1) a nominative ATU, delivered for a single, named patient at the request and under the responsibility of the prescribing physician; and (2) a cohort ATU, provided for a group or subgroup of patients at the request of the holder of the licensing rights (e.g., the manufacturer). Patients must be treated and monitored according to criteria fully defined in a protocol for therapeutic use and information collection (33, 34).

Medicinal products with an ATU can be prescribed only in hospitals, and they are either administered to inpatients or dispensed to outpatients by hospital pharmacies. ATUs for inpatient use are funded through a specific financial allowance under Teaching, Research, Reference and Innovation Missions (Missions d'Enseignement, de Recherche, de Référence et d'Innovation, or MERRI), a subgroup of financial allowance of Missions of General Interest and Support to Contracting (Missions d'Intérêt Général et d'Aide à la Contractualisation, or MIGAC). ATU products for outpatient dispensation are funded through the reassigned list (‘Liste de rétrocession’). The price of medicinal products with an ATU is set freely by the manufacturer, and a maximum price is submitted to the CEPS by the pharmaceutical company. If the negotiated price, following market authorisation and completion of the pricing and reimbursement process at the national level, is lower than the ATU price, manufacturers are required to pay back the total revenue difference between the ATU price and the actual post-launch reimbursed price for the whole period between the ATU authorisation and price publication in the French gazette. Products with an ATU are reimbursed 100% by the National Health Insurance during the ATU validity period, even if the marketing authorisation is granted before the end of this period (19, 35–38).

Innovative products in development may also benefit from funding via MERRI through three main research programmes: (1) the Translational Research Program (Programme de Recherche Translationnelle, or PRT); (2) the Hospital Program of Clinical Research (Programme Hospitalier de Recherche Clinique, or PHRC); and (3) the Health Economic Research Program (Programme de Recherche Médico-Economique, or PRME). The PRT enables funding of specific collaborative research on specific scientific topics between explorative research and clinical research, and results from this research should support the development of new hypotheses to be tested further in clinical research. The PHRC enables funding of clinical research projects that have a direct impact on patient management. Research areas of priority for the PRT and PHRC are defined by the Ministry of Health and described in the appendix of the annual circular of project calls from the General Directorate of Health Care Supply (Direction Générale de l'Offre de Soins, or DGOS). PRME enables funding of comparative health economic studies along two research axes: a health innovation axis to determine the efficiency of innovative health technologies and their budget impact in the French context, and a healthcare pathway axis to identify the most efficient strategy for patient care. To be eligible, the efficacy and safety of innovative technologies must be validated (i.e., with a CE mark for medical devices or marketing authorisation or temporary authorisation for use for medicines). PRME results are expected to facilitate and speed up the assessment of innovative technologies by the HAS. Applications for all these research programmes need to be submitted by health practitioners (39, 40).

Moreover, medical procedures that are in the validation process (outside nomenclature) can also be funded via MERRI (40).

According to the social security funding law for 2009 (41) and for 2015 (42), and the application decree issued in February 2015 (43), all medical devices and medical procedures that are considered innovative in terms of clinical or health economic benefit can apply for the ‘forfait innovation’ (Article L165-1-1 of Social Security Code (44)), for which temporary funding is conditional on a clinical or health economic study. The technology will be acknowledged as innovative if it meets the following four criteria: (1) novelty; (2) early development stage for patient access with insufficient data, and no part of any past or current public funding; (3) a safety profile assessed from available clinical trials; and (4) an important expected clinical benefit to satisfy an unmet need, or a significant health economic benefit with (at least) clinical non-inferiority. The decision to approve treatments for the ‘forfait innovation’ lies with the Ministry of Health and is published in a specific order (or Arrêté) which specifies: lump sum per patient (covering medical procedure, medical device cost, and hospitalisation costs, if applicable), number of eligible patients, coverage duration, specific conditions of use, list of healthcare institutions where the technology is covered, and modalities for lump sum allocation to healthcare institutions (45).

#### ATMPs prepared on a non-routine basis (hospital exemptions)

Hospital exemptions are authorised by the ANSM and can be delivered only in specific authorised centres. There is currently no published list of these therapies in France, however, experts’ feedback – at the time of conducting this research – indicates that some dossiers have been submitted to the ANSM to get this status, but are still pending approval. When experts were challenged about the impact these exemptions may have on marketing authorisation applications, it was emphasised that in France, it is not expected that licenses for ATMPs prepared on a non-routine basis would be maintained if an ATMP was centrally approved for the same indication in the same patient population.

**Fig. 4:**
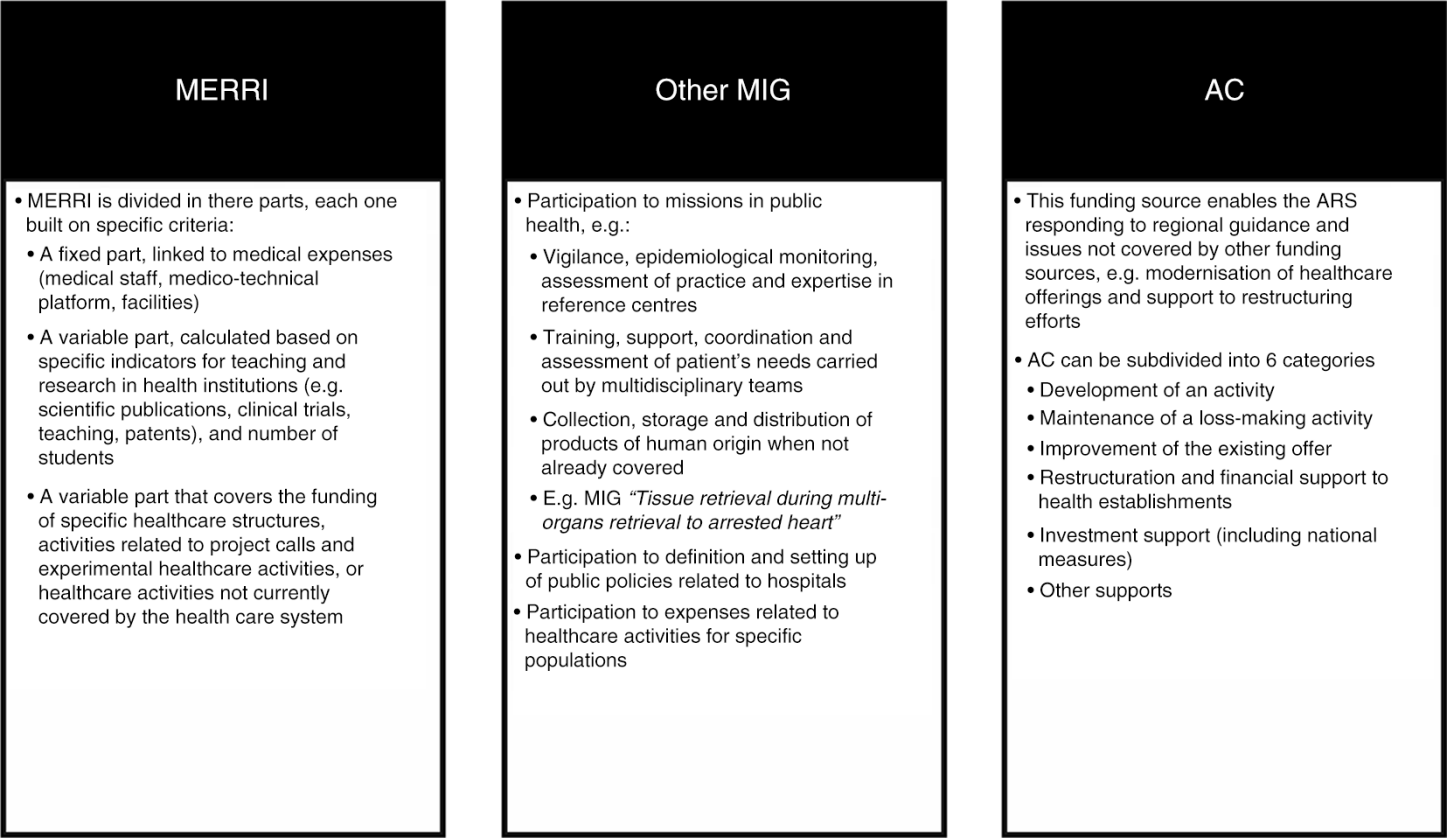
Funding sources outside the DRG system (outside T2A system) (46–48). AC: Support to Contracting; MERRI: Teaching, Research, Reference and Innovation Missions; MIG: Missions of General Interest.

With regard to the funding for these products, the most likely option is funding outside the DRG through MIGACs. Funding sources outside of the DRG have been set up to support non-quantifiable activities that cannot be charged to health insurance. MIGACs are part of a specific funding system that does not come under the activity-based system of payment. The Ministry of Health defines the list of ‘missions of general interest’, and the overall MIGAC target is set at the national level and then allocated on a regional basis by the Regional Health Agencies (ARS). MIGACs are subdivided into two categories: MIGs, including the MERRI and other MIGs, and Support to Contracting (Aides à la Contractualisation, or AC) (46) (Fig. 4).

Within the Ministry of Health, three directorates are involved in allocating MIGAC funding: (1) the General Directorate of Health Care Supply (DGOS), which is the main stakeholder managing the sums allocated to each region and relationships with the ARS; (2) the Social Security Directorate (Direction de la Sécurité Sociale, or DSS), which discusses the targeted amount voted each year with the DGOS (Social Security Funding Law) and is one of the decision makers in the event of a suggestion to create or remove a MIG; and (3) the Health General Directorate (Direction Générale de la Santé, or DGS), which suggests new categories of MIGAC funding (49).

#### Minimally manipulated cells (cell preparations)

Each year, the Biomedicine Agency publishes the funding modalities of healthcare institutions for retrieval and transplantation of organs, tissues, and haematopoietic stem cells. Four different funding processes are applicable, depending on the type of activity: (1) inclusion in the DRG; (2) inclusion in the LPPR (as a medical device); (3) annual lump sum provided by ARS; and (4) financial allowance under a MIG (50).

The current DRG tariffs include funding specifically for the following tissue allografts: corneal tissue and amniotic membranes, skin, heart valves, tendons, and ligaments. These tariffs include funding for tissue retrieval and donor serological tests, transport, and tissue bank costs (processing, storage, distribution, and traceability). Retrieval of haematopoietic stem cells is also covered through DRG tariffs if it is performed on living donors during a hospitalisation (50).

Tissue allografts are currently funded outside the DRG when the grafts are bones or vessels. Similarly, this funding covers the costs related to tissue retrieval and donor serological tests, transport, and tissue bank costs (processing, storage, distribution, and traceability) (50).

Annual lump sums for coordination of organ and tissue retrieval (CPO) as well as for haematopoietic stem cell graft (FAG) are provided by the ARS to the authorised healthcare institutions. The yearly lump sum amount is based on the activity of the previous year. The hospital tissue banks benefit from a financial allowance under a MIG entitled ‘Tissue retrieval during multi-organs retrieval to arrested heart’, to support their activities and organisation at a global level (50).

### Cell therapies available in France

So far, four cell-based medical products have been granted a centralised marketing authorisation within the EU: ChondroCelect^®^ (51) (autologous cartilage cells), MACI^®^ (52) (autologous cultured chondrocytes), Provenge^®^ (53) (autologous peripheral-blood mononuclear cells), and Holoclar^®^ (9) (autologous human corneal epithelial cells) (Table 2). However, since then, the marketing authorisations for MACI^®^ and Provenge^®^ were suspended (in 2014 and 2015, respectively) for commercial reasons^17^ (55, 56).

**Table 2:** Cell-based medicinal products with EU centralised marketing authorisation and CT opinion status (9, 51–54)

Brand name	Manufacturer	Active substance	Therapeutic area	Indication	Pharmaceutical form	Centralised MA date	CT opinion
ChondroCelect^®^	TiGenix NV	Characterised viable autologous cartilage cells expanded *ex vivo* expressing specific marker proteins	Orthopaedics	Repair of single symptomatic cartilage defects of the femoral condyle of the knee	Implantation suspension	05 October 2009	Insufficient actual benefit
MACI^®^	Genzyme Europe B.V.	Implant containing matrix applied characterised autologous cultured chondrocyte	Orthopaedics	Repair of symptomatic, full-thickness cartilage defects of the knee	Implantation matrix	27 June 2013 SUSPENDED in 2014	Not evaluated
Provenge^®^	Dendreon UK Ltd	Autologous peripheral blood mononuclear cells activated with PAP-GM-CSF (Sipuleucel-T)	Oncology	Treatment of asymptomatic or minimally symptomatic metastatic (non-visceral) castrate-resistant prostate cancer in male adults in whom chemotherapy is not yet clinically indicated	Dispersion for infusion	06 September 2013 WITHDRAWN in 2015	Not evaluated
Holoclar^®^	Chiesi Farmaceutici S.p.A.	Ex vivo expanded autologous human corneal epithelial cells containing stem cells	Ophthalmology	Treatment of adult patients with moderate to severe limbal stem cell deficiency (defined by the presence of superficial corneal neovascularisation in at least two corneal quadrants, with central corneal involvement, and severely impaired visual acuity), unilateral or bilateral, due to physical or chemical ocular burns	Living tissue equivalent, Transparent, circular sheet	17 February 2015 CONDITIONAL APPROVAL	Not evaluated

MA: marketing authorisation; CT: Transparency Committee.

Only ChondroCelect^®^ (indicated for the repair of single symptomatic cartilage defects of the femoral condyle of the knee) has been assessed by the CT, but reimbursement was not granted. A first assessment was performed by the CT in 2010, and ChondroCelect^®^ was recognised as an innovative biotechnology, but the actual benefit (Service Médical Rendu, or SMR) was considered as insufficient (11). The CT was unable to evaluate the therapeutic benefit of ChondroCelect^®^, particularly in preventing the onset of osteoarthritis in the long term. The committee highlighted that clinical efficacy had not been established in the clinical trials^18^ and that the medicinal product was unlikely to provide an additional benefit to the identified public health need. The Committee also estimated that (1) the transferability of results from the pivotal study to clinical practice was associated with significant uncertainty (due to exclusion criteria); and (2) the use of ChondroCelect^®^ could potentially have a negative financial impact on the healthcare system, as the treatment involved at least two hospitalisations, whereas the comparative technique (microfracture) required just one. The manufacturer resubmitted additional data providing longer term information that were assessed by the CT in 2013 (12). Nonetheless, the CT drew the same conclusions, and argued that data did not demonstrate conclusively the treatment efficacy and also showed a larger number of adverse effects versus the comparator (microfracture).

With regard to the other categories of cell therapies, the ANSM does not publish any specific list of ATMPs prepared on a non-routine basis or of minimally manipulated cells (cell preparations); only the list of healthcare institutions authorised to handle such products is available (57, 58).

## Market access of cell therapies in France: Roadmap (Fig. 5)

**Fig. 5:**
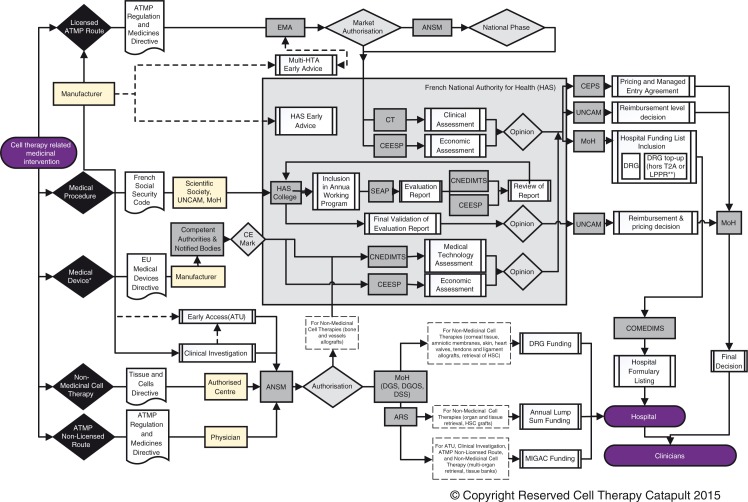
Market access of cell therapies in France: roadmap. *Can be associated with drugs/medical procedure. In this case, drug/medical procedure processes apply. **Funding framework for medical devices and non-medicinal cell therapies (used both for ambulatory care and inpatient DRG top-up payments). ANSM: National Agency for the Safety of Medicine and Health Products; ARS: Regional Health Agencies; ATMP: Advanced Therapy Medicinal Product; COMEDIMS: Committee for Medicines and Sterile Medical Devices; CEESP: Economic and Public Health Assessment Committee; CEPS: Economic Committee for Health Care Products; CNEDIMTS: National Committee for the Evaluation of Medical Devices and Health Technologies; CT: Transparency Committee; DGOS: General Directorate of Health Care Supply; DGS: Health General Direction; DRG: Diagnosis Related Group; DSS: Social Security Direction; EMA: European Medicines Agency; HAS: French National Authority for Health; MIGAC: Missions of General Interest and Support to Contracting; MoH: Ministry of Health; SEAP: Department of Medical Procedures Assessment; UNCAM: National Union of Health Insurers.

## Discussion

Cell therapies represent a heterogeneous group of therapies with different regulatory classifications that determine the subsequent market access pathways in France.

Although healthcare delivery in France is undergoing decentralisation, the funding decisions for therapeutic interventions remain highly centralised. French authorities have been pro-active in ensuring a clear classification and a regulatory path for all cell therapies; simultaneously, as seen in the past, the funding process remains reactive and progresses in a pragmatic way, where changes in rules and processes are typically based on experience and adjusted as needed. Therefore, more specific funding options could emerge as the number of cell therapies increases and the authorities face the need to structure and stabilise funding. One possible future development is that specific healthcare providers (e.g., hospitals) could be appointed as reference centres for cell therapies indicated in rare and severe diseases, and funded via a lump sum covering the provision of the procedure and therapy costs through MIGACs.

In France, assessment of cell therapies classified as ATMPs is similar to that for other innovative medicines; the importance of robust clinical study design, comparative evidence, and transferability of clinical study results in clinical practice remains. More so than for pharmaceuticals, payers are expected to pay specific attention to the long-term safety and efficacy of cell therapies, which will necessitate collection of long-term real-world data. For highly innovative therapies with substantial improvements in outcomes, the CT is already open to flexible designs and is using a pragmatic approach in its assessment; data quality, clinical relevance, and effect size will determine the degree of this flexibility. France has always shown considerable willingness to reward innovation and to provide patient access, and the relatively high prices and broad access to oncology products (as compared to other European countries) are testimony to this practice. It is unlikely that therapies that are indeed innovative from a patient outcome perspective will be denied reimbursement.

The main challenges facing cell therapies relate to price setting, as high manufacturing costs dictate premium prices in order to be commercially viable. Although many cell therapies are anticipated to provide long-term benefits following a single treatment, it will be difficult for payers to reward such therapies for their full benefits upfront, because of uncertainty over long-term performance, and also because of the budget impact concerns.

Managed entry agreements are expected to be applied to many of these therapies, with pricing and reimbursement being conditional upon evidence development and payments being based on actual savings or benefits accrued over a defined period. Also, health economics will increasingly be applied in order to support the translation of clinical outcomes and resource implications to reimbursed price potential, while accounting for affordability.

For hospital exemptions and minimally manipulated cells, the market access and funding situation is quite complex and, in many cases, relies on non-permanent funding options (e.g., hors T2A or LPPR) except when a sufficient DRG tariff is already available. This highlights the need for a full understanding of the various temporary funding opportunities that occur early in a product lifecycle and the adoption of a stepwise approach to secure permanent funding.

It is important to note that the funding arrangements for minimally manipulated cell therapies described here might change in the future. As highly innovative minimally manipulated cell therapies are emerging with the potential to deliver substantial incremental benefits to the patient and the healthcare system over existing therapeutic alternatives, the way these will be assessed for reimbursement and funding in the future could mirror the licensed ATMP processes.

Although it is not expected that hospital exemptions will replace centrally approved products, they might be used tactically by payers to delay market access of centrally approved products that come in at a higher price. This could potentially be used in price negotiations by arguing that patients have access to a therapeutic option, thus possibly delaying the time to market and reducing the reimbursed price potential.

The pathway for medical procedure funding is not straightforward, and it is associated with substantial uncertainty. The HAS receives more medical procedure applications than they can review, and a prioritisation is performed that in certain cases can delay review by several years. In addition, the medical procedure review process does not allow any price negotiation. The assessment and funding processes for cell therapies that include new procedures are expected to be two-fold (i.e., separately for the therapy and the procedure).

### Conclusion and strategic recommendations for manufacturers

Cell therapy is a complex and heterogeneous category of medical interventions, meaning several funding options and market access pathways apply; it is therefore vital for manufacturers to have a clear understanding of the relevant market access processes in France. This complexity, along with the reactive way in which funding is updated, highlights the importance for the industry to communicate and establish relationships and partnerships with key stakeholders, such as key opinion leaders in the relevant fields (physicians and researchers); national-, regional-, and local-level payers; hospital management; patient associations; administration; and key institutions.

The licensed ATMP route is the most attractive, as it provides data and market exclusivity (under regulatory legislation) in addition to having a highly standardised and therefore more predictable market access pathway. For ATMPs indicated in rare and/or severe conditions and demonstrating a substantial clinical benefit, France is expected to favour early access to such therapies; in other cases, stringent evidence requirements are expected. Reimbursed early access exists in France through the ATU scheme, and is of high strategic importance as it can reduce time to market by up to 2 years.

Obtaining funding on top of DRG tariffs is instrumental for access and uptake of ATMPs used in hospitals. Manufacturers of cell therapies with a substantial procedure component need to be aware that the medical procedure pathway remains extremely time-consuming and uncertain, and this is unlikely to improve in the coming years. Therefore, it is highly important to carefully map existing tariffs and understand how any funding gaps can be addressed, to minimise the risk of undergoing a lengthy procedure assessment.

Furthermore, opportunities exist to obtain ad-hoc funding for evidence generation regarding cell therapy use and real-life outcomes. It should be noted that this is limited to treatments with particularly promising therapeutic features, and would be less likely to be applied in cases where there are only small incremental improvements in clinical benefit. Such evidence generation activities provide an opportunity for the manufacturer to gain experience in the French healthcare system and also engage with key opinion leaders and achieve support.

Very limited experience is available in terms of cell therapies that have gone through the French HTA for pricing and reimbursement, meaning that great uncertainties remain concerning their assessment. Therefore, manufacturers should enter into dialogue with HTA agencies at an early stage to reduce uncertainty and mitigate these risks.

## Supplementary Material

Market access pathways for cell therapies in France

## Footnotes

1 Other directives specifically cover blood and blood products (other than haematopoietic progenitor cells) and human organs. Available from: www.ec.europa.eu/health/blood_tissues_organs/key_documents/index_en.htm#anchor5

2 ‘Somatic cell therapy medicinal product means a biological medicinal product which has the following characteristics: 1) contains or consists of cells or tissues that have been subject to substantial manipulation so that biological characteristics, physiological functions or structural properties relevant for the intended clinical use have been altered, or of cells or tissues that are not intended to be used for the same essential function(s) in the recipient and the donor; 2) is presented as having properties for, or is used in or administered to human beings with a view to treating, preventing or diagnosing a disease through the pharmacological, immunological or metabolic action of its cells or tissues’.

3 ‘Tissue engineered product means a product that: – contains or consists of engineered cells or tissues, and – is presented as having properties for, or is used in or administered to human beings with a view to regenerating, repairing or replacing a human tissue’.

4 ‘Combined ATMT means an ATMT that fulfils the following conditions: – it must incorporate, as an integral part of the product, one or more medical devices within the meaning of Article 1(2)(a) of Directive 93/42/EEC or one or more active implantable medical devices within the meaning of Article 1(2)(c) of Directive 90/385/EEC, and – its cellular or tissue part must contain viable cells or tissues, or – its cellular or tissue part containing non-viable cells or tissues must be liable to act upon the human body with action that can be considered as primary to that of the devices referred to’. An example cited in the EMA reflection paper on classification of ATMTs: ‘The expanded autologous chondrocytes seeded onto a collagen membrane and administered, fixed on this membrane, into the joint cartilage lesion. The primary action of the combined product is given by the viable cells that repair the damaged tissue, while the medical device part is a tool that is needed to retain the cells physically to the cartilage defect’.

5
www.sante.gouv.fr/

6
www.ansm.sante.fr/

7
www.has-sante.fr/portail/jcms/fc_1249588/fr/accueil

8
www.ameli.fr/

9
www.agence-biomedecine.fr/

10
www.legifrance.gouv.fr/

11 The ‘blue box’ is a boxed area included in the labelling, with a blue border, aimed at containing information specific to each Member State.

12 Drugs awarded ASMR IV are also eligible for a fast-track procedure, provided that:

- Daily treatment cost does not exceed that of the comparator or, if treatment costs are higher, cost savings for the health insurance system are demonstrated with the new drug.
- The drug is not designed to replace a genericised product, or one that will soon face generic competition.

13 Pharmaceutical companies that invest in research and development or production in France, expand their head count, and contribute to the national economy might benefit from higher consideration from the CT. Small companies can also benefit from a more lenient attitude.

14 Expected use of the drug in the considered therapeutic indication, estimate of prescription frequency during hospitalisation, average treatment cost estimate during hospitalisation, and application of the principle of treatment equity.

15 CNEDiMTS assessment every 5 years maximum of generic descriptions via annual programme of review (each year, the list of products to be re-examined in order to renew inclusion is fixed by decree and published in the French official gazette).

16 When the impact on health insurance payments, public health requirements, and the control and/or the difficulty of defining minimal technical specifications require specific monitoring of the product.

17 In 2014, the marketing authorisation holder closed the EU manufacturing site for commercial reasons, and the marketing authorisation of MACI has been suspended until a new manufacturing site is registered in the EU. On 6 May 2015, the European Commission withdrew the marketing authorisation for Provenge at the request of the marketing authorisation holder for commercial reasons.

18 Available data from a single randomised, open comparative study in a limited number of patients (112 patients analysed, and 51 treated with ChondroCelect) and based on a composite clinical criterion.
