# Supplementary material for: Market access pathways for cell therapies in France
Source: J Mark Access Health Policy. 2015 Nov 18;3:10.3402/jmahp.v3.29094. doi: 10.3402/jmahp.v3.29094 (PMC4802688; doi:10.3402/jmahp.v3.29094)
Supplement: Market access pathways for cell therapies in France [file JMAHP-3-29094-s001.docx]

**Supplement 1-Discussion guide for expert’s interviews**

# Background on Cell Therapies in France

- 1. Definitions

In France, cell therapies can be classified in 3 categories of products regulated according to 3 different frameworks.

Example: ChondroCelect^®^ (characterised viable autologous cartilage cells expanded ex vivo expressing specific marker proteins) (see below)

Example: haematopoietic stem cells for hematopoietic reconstitution

- 1. ATMPs Approvals ***(somatic cell therapy medicinal product, tissue engineered product)***

| Brand name | Manufacturer | | Nature | | Therapeutic area | | Indication | | Pharmaceutical form | | Centralised MA date | | CT decision |
| --- | --- | --- | --- | --- | --- | --- | --- | --- | --- | --- | --- | --- | --- |
| ChondroCelect^®^ | TiGenix NV | Characterised viable autologous cartilage cells expanded ex vivo expressing specific marker proteins | | Regenerative medicine (orthopaedics) | | Repair of single symptomatic cartilage defects of the femoral condyle of the knee | | Implantation suspension | | 05/OCT/2009 | | Insufficient actual benefit | |
| Maci^®^ | Genzyme Europe B.V. | Implant containing matrix applied characterised autologous cultured chondrocyte | | Regenerative medicine (orthopaedics) | | Repair of symptomatic, full-thickness cartilage defects of the knee | | Implantation matrix | | 27/JUN/2013 | | Not yet evaluated | |
| Provenge^®^ | Dendreon UK Ltd | Autologous peripheral blood mononuclear cells activated with PAP-GM-CSF (Sipuleucel-T). | | Oncology | | Indicated for treatment of asymptomatic or minimally symptomatic metastatic (non-visceral) castrate resistant prostate cancer in male adults in whom chemotherapy is not yet clinically indicated | | Dispersion for infusion | | 06/SEP/2013 | | Not yet evaluated | |

# General Considerations

| 1. What is your general perspective (current and future) on cell therapies in France? |  |
| --- | --- |

# Cell Therapies available in France *(All)*

| 1. Does it exist a published list of cell preparations and ATMPs prepared on a non-routine basis? | Yes  No  If Yes, please specify |
| --- | --- |

# Funding Options

| - Several funding options were identified for cell therapies:   - Inclusion on the list of medicines refundable by National Health Insurance   - Inclusion in Diagnosis Related Group (DRG)     - Tissue allograft (Corneal tissue and amniotic membranes, Skin, Heart valves, Tendons and ligaments, Retrieval of hematopoeitic stem cells if performed on living donors during an hospitalization)   - Inclusion on the list of products and services qualifying for reimbursement (LPPR)     - Tissue allograft (Bone, Vessels)   - Annual lump sum provided by Regional Health Agencies (ARS)     - Annual lump sum (CPO) provided by ARS for hospital coordination of organ and tissue retrieval     - Annual lump sum (FAG) provided by ARS for hematopoietic stem cell graft   - Financial allowance under MIG (Missions of General Interest):     - Via MERRI funding (Teaching, Research, Reference and Innovation Missions) through various programs       - PRT (Translational Research Program)       - PHRC (Hospital program of Clinical Research)       - PRME (Medico-Economic Research Program)       - Medical procedures in validation process (outside nomenclature)     - Via other MIG: «*Tissue retrieval during multi-organs retrieval to arrested heart* » to support at a global level the activities and organisation of tissue hospital banks   - Financial allowance under AC (Support to Contracting) |
| --- |

| 1. Could you please specify if other funding options are available for cell therapies? | Yes  No  Please specify  3a.Especially, does it exist different funding pathways for ATMPs prepared on a non-routine basis?  Yes  No  Please specify  3b.Are specific funding options in place in your establishment?  Yes  No  Please specify |
| --- | --- |

| 4. Could you please describe the process for inclusion/DRG creation related to high cost therapies such as cell therapies (*which might include drug/device and associated procedure*)? | 4a. What is the process and what are the timelines?  4b.Who are the decision-makers?  4c.How is budget allocated and how is expenditure anticipated?  4d.What are monitoring measures put in place when the therapies are included into DRG and their impacts?  4e.Would you have some examples of cell therapy products recently included into DRG (new DRG and inclusion into existing DRG)? |
| --- | --- |

| 5.Could you please describe the process for financial allowance under MIG and AC related to high cost therapies such as cell therapies (*which might include drug/device and associated procedure*)? | 5a. What is the process (including renewals) and what are the timelines?  5b.Who are the decision-makers?  5c.How is budget allocated and how is expenditure anticipated?  5d.What are monitoring measures put in place when financial allowance has been granted to these therapies and their impacts?  5e. Would you have some examples of cell therapy products which recently benefit from these funding options? |
| --- | --- |
| 6. How are the financial and clinical aspects weighted against each other in the funding decision? |  |
| 7. In the absence of licensed therapeutic alternatives, so-called hospital exemption might be an option to provide patients the possibility to benefit from a custom-made, innovative individual treatment. To qualify for this hospital exemption, the ATMPs should meet all the following criteria:  - Preparation on a non-routine basis  -Preparation according to specific quality standards  - Use within France  - Use in a hospital  -Use under the exclusive responsibility of a medical practitioner  -Comply with an individual medical prescription for a custom-made product for an individual patient  National exempted products have different and often less stringent criteria and rules as those applied for licensed ATMPs.  Licensed ATMP are expected to be higher price options than hospital exemptions. | |
| 7a. If a licensed ATMP is launched for the same indication in the same patient population where a cheaper hospital exemption exists, which option would you select? | Hospital exemption  Licensed ATMP  Please specify your selection criteria |

| 8. Regarding potential cost savings associated with a cell therapy, are there any types of savings or improvements you value more than others?  *Please rate importance of these items on a scale from 1 to 5 where 1 represents the lowest level of importance and 5 represents the highest level importance*  *(to address also this point with HTA/payers)* | 8a.Reduction in use of other drugs or devices?     \| 1 \| 2 \| 3 \| 4 \| 5 \| \| --- \| --- \| --- \| --- \| --- \|   Please elaborate  8b.Reduction in number of hospitalizations?     \| 1 \| 2 \| 3 \| 4 \| 5 \| \| --- \| --- \| --- \| --- \| --- \|   Please elaborate  8c.Reduction in hospital length of stay?     \| 1 \| 2 \| 3 \| 4 \| 5 \| \| --- \| --- \| --- \| --- \| --- \|   Please elaborate  8d.Reduction in rehabilitation?     \| 1 \| 2 \| 3 \| 4 \| 5 \| \| --- \| --- \| --- \| --- \| --- \|   Please elaborate  8e.Ability to go back to work?     \| 1 \| 2 \| 3 \| 4 \| 5 \| \| --- \| --- \| --- \| --- \| --- \|   Please elaborate  8f.Other: Please specify |
| --- | --- | --- | --- | --- | --- | --- | --- | --- | --- | --- | --- | --- | --- | --- | --- | --- | --- | --- | --- | --- | --- | --- | --- | --- | --- | --- |

| 9.Do/Did you experience some issues related to funding of cell therapies? | Yes  No  Please specify |
| --- | --- |
| 10.What are your perspectives on budgets to be allocated and funding options for cell therapies in the 5 years to come? |  |

# Clinical Trial Design *(Physicians and HTA perspective)*

| - Cell therapies have huge potential for curing many chronic disabling diseases and injuries such as Alzheimer’s disease, Parkinson’s disease, cancer, muscular dystrophy, skin in burns, and cartilage injuries - Example 1/Parkinson’s disease: To reverse the damage to nerve cells in the brain by brain transplantation of embryonic stem cells directed to make dopamine-producing neurons - Example 2/Oncology: Adoptive cell transfer involving engineering patient’s own immune cells to recognize and kills cancer cells with a specific antigen on their surface (CAR T-Cell therapy) - Example 3/Congestive heart failure: To repair damaged tissue and improving heart function and patient clinical outcomes by intramyocardial injection of cardiopoietic [stem cells](http://www.c3bs.com/en/glossary#Stem_cells) - Considering the wide range of diseases which might be treated by this type of therapies, we would like to have your (“conceptual”) perspectives on the following questions |
| --- |

| 11.Would you have any specific requirements in terms of efficacy or safety of cell therapy products? |  |
| --- | --- |
| 12. Considering specificities of these therapies (e.g. some of these therapies administered through invasive procedures, disease-modifying therapies versus currently symptomatic comparative therapies) what are your perspectives on clinical trial designs? | Yes  No  Please specify |
| 13. Would you expect real world data studies becoming a rule for all of these therapies? |  |
| 14. How do you determine whether a treatment qualifies as a drug treatment or a procedure? |  |

# Perspective on P&R *(HTA, payers*)

| - About 300 ATMPs are currently in clinical development   - About 70% are cell therapies - These therapies that might cure chronic and/or severe diseases, might add substantial burden on the healthcare system and question their sustainability - Pricing and reimbursement decisions seem to be more and more driven by budget constraint   - Very recently, social security funding bill for 2015 introduced proposal regarding hepatitis C therapies to contain potential impact on healthcare budget of costly medicines (Solvadi®) through creation of a progressive contribution mechanism for hepatitis C drug if annual turnover exceeds a threshold set by law   - Innovative managed entry agreements might also be more and more considered in the future     - Eg. Pay for performance split over years as modelled below for heart failure and Parkinson’s disease *(Toumi M., Kornfeld A., Charaf A. Impact of Advanced Therapy Medicinal Products cost on public healthcare budgets - ISPOR 19th Annual International Meeting, Montreal, QC, Canada, May 31- June 4, 2014. PHP57)*  \| Estimated yearly direct costs per patient \| \| \| \| \| --- \| --- \| --- \| --- \| \| **Pathology** \| **ATMP (single payment)** \| **ATMP (annual payment)** \| **Standard of care** \| \| **Heart failure** \| €165,590 \| €16,122 \| €1,141 \| \| **Parkinson’s disease** \| €154,257 \| €13,351 \| €7,659 \| |
| --- | --- | --- | --- | --- | --- | --- | --- | --- | --- | --- | --- | --- | --- | --- | --- | --- |

| 15. What are your perspectives on the challenges faced with cell therapies in terms of P&R? |  |
| --- | --- |
| 16.Do you anticipate specific managed entry agreements schemes for cell therapies? If yes, which types of agreements, under which conditions and their potential challenges? | Yes  No  Please specify |
| 17.Do you anticipate any specific requirements in terms of health economics data? | Yes  No  Please specify |
| 18.Do you anticipate an important impact of health economic assessment in price setting? | Yes  No  Please specify |

# Conclusions

| 19. Do you have any specific recommendations for cell therapies manufacturers for successful market access in France? |  |
| --- | --- |

***Thank you very much for your time***
